# Comparison of various formulae for estimating low-density lipoprotein cholesterol by a combination of ages and genders in Taiwanese adults

**DOI:** 10.1186/1471-2261-14-113

**Published:** 2014-09-02

**Authors:** Chung-Huang Tsai, Hsin-Hung Wu, Shao-Jen Weng

**Affiliations:** Department of Family Medicine, Chung-Kang Branch, Cheng Ching General Hospital, Taichung, 40799 Taiwan; Department of Business Administration, National Changhua University of Education, Changhua, 500 Taiwan; Department of Industrial Engineering and Enterprise Information, Tunghai University, Taichung, 40704 Taiwan

**Keywords:** Low-density lipoprotein cholesterol, Residual cholesterol, Friedewald formula, Triglyceride

## Abstract

**Background:**

The accuracy and precision of the Friedewald formula for estimating low-density lipoprotein cholesterol (LDL-C) is questionable. Although other formulae have been developed, only a few studies compare them. Thus, we compared the efficiencies of various formulae, based on the age and gender of adults, to determine which ones yield more accurate estimations in terms of mean squared error, and which formulae underestimated and overestimated LDL-C performance.

**Methods:**

This study compares various formulae in terms of mean squared error (MSE), as well as underestimation and overestimation of LDL-C concentrations, using subjects of various ages and both genders. Six groups were examined in this study based on age and gender: males 20–44 years old, 45–64, and 65 and above, and females in the same three age ranges.

**Results:**

The results show that the Friedewald formula has relatively low accuracy, and while its performance among older (aged 45 and above) women with triglyceride concentrations ≤ 400 mg/dL is better than that with other groups, it is still more inaccurate than the other formulae. In terms of prediction errors and mean squared errors, Tsai’s formula (TF) and a calibrated TF provide the most accurate results with regard to the LDL-C concentration. Moreover, based on a cross-validation of age and gender, these two formulae provide highly accurate results for the LDL-C concentrations of all the studied groups, except for women aged 20–44 years.

**Conclusions:**

Based on the experimental results, this study provides a set of benchmarks for the formulae used in LDL-C tests when considering the factors of age and gender. Therefore, it is a valuable method for providing formula benchmarking.

## Background

Medical research and clinical trials have shown that the low-density lipoprotein cholesterol (LDL-C) concentration is causally related to an increased risk of coronary artery disease [1, 2]. In addition, a report by the National Cholesterol Education Program Adult Treatment Panel III notes that the level of LDL-C is the primary variable that is used to predict cardiovascular disease [1]. One well-known formula for calculating this, the Friedewald formula (FF), is of doubtful accuracy and precision, and thus other approaches have been developed, such as DeLong’s formula (DF) [3, 4], Teerakanchana’s multiple regression (MR) [5], Balal’s formula (BF), which is derived from the FF [6], Tsai’s formula (TF) [7], calibrated from TF (CTF) [8], and Tsai’s multiple regression (TMR) [8]. All of these formulae measure the LDL-C concentrations based on total cholesterol (TC), high-density lipoprotein cholesterol (HDL-C), and triglyceride (TG) concentrations [9–12]. Several studies compare the various methods used to assess the LDL-C concentration, and this is likely due to rising healthcare expenditures as well as an increasing demand for quality healthcare. It is thus highly desirable to identify an accurate, a cost-effective method to determine the LDL-C concentration.

Most clinical trials employ the FF [3], which uses TC, HDL-C, and TG to measure the levels of LDL-C [5]; thus, it can be applied to the clinical treatment and prevention of atherosclerotic disease [6, 8]. However, the FF has produced inaccurate results in some cases, and it is not recommended for use in the presence of hypertriglyceridemia (>400 mg/dL) or type III hyperlipoproteinemia [13]. This method also tends to underestimate LDL-C concentrations [6, 14–18] when the triglyceride concentration is normal [19, 20] or less than 400 mg/dL [4, 6, 21, 22]. Balal et al. [6] thus revised the FF for use with renal transplant recipients by considering those with TG concentrations lower than 400 mg/dL to calculate LDL-C levels. Teerakanchana et al. [5] developed a multiple regression formula by using a multiple linear regression model to test different data sets. Tsai et al. [8] further took into account residual cholesterol (RC), which consists of high-density lipoprotein cholesterol (HDL-C), and revised the FF by using TG = 1/8 instead of TG = 1/5, which represents very-low-density lipoprotein cholesterol (VLDL-C).

LDL-C can now be measured directly using advanced technologies, and while the time and cost of these technologies continue to decrease, their costs remain relatively high compared to using formulae to produce estimates. LDL-C concentration may thus be determined in hospitals, at least in part, through best practice measures, and TMR is a valuable method for providing benchmarking data [8]. However, no studies to date have explored the use of formulae to estimate LDL-C concentration among subjects of different ages and genders. Measuring LDL-C without considering age and gender may produce misleading results, because one formula may perform well with one age group or gender, but perform poorly with others. This study thus compares all seven formulae shown in Table 1 in terms of mean squared error (MSE), as well as underestimation and overestimation of LDL-C concentrations, using subjects of various ages and both genders.

**Table 1:** **Comparison of seven LDL-C formulae**

Author	Formula
Friedewald et al. [3]	FF:
	LDL-C = TC- (HDL-C) - (TG/5)
Balal et al. [6]	BF:
	LDL-C =8.018 + 0.99(LDL-C predicted by FF)
Delong et al. [4]	DF:
	LDL-C = TC- (HDL-C)- 0.16TG
Teerakanchanna et al. [5]	MR:
	LDL-C = 0.910TC - 0.634(HDL-C) - 0.111TG - 6.755
Tsai et al. [7]	TF:
	LDL-C = TC- (HDL-C) - (TG/8)
Tsai et al. [8]	CTF:
	LDL-C =0.276 + 0.997(LDL-C predicted by TF)
Tsai et al. [8]	TMR:
	LDL-C =0.988TC - 0.853(HDL-C) - 0.107TG - 8.703

Note: *TC* total cholesterol, *LDL-C* low density lipoprotein-cholesterol, *HDL-C* high density lipoprotein-cholesterol, *TG* triglyceride.

## Methods

### Study population

The data used in this study was collected from Cheng Ching General Hospital in Taiwan in 2011, with 3,532 valid samples obtained for measurement of LDL-C concentration. All subjects were 20 to 95 years old, with TG concentrations ≤ 400 mg/dL (n = 3,395; 96.1%) and > 400 mg/dL (n = 137; 3.9%). The subjects were classified into three groups according to age, i.e., younger (20–44 years old), middle-aged (45–64 years old), and elderly (65 years old and above). The subjects’ basic information with regard to TC, HDL-C, LDL-C, and TG is summarized in Table 2. The maximum and minimum of TG are 1252 and 22 mg/dL. Moreover, the maximum values of TC, HDL-C, and LDL-C are 569, 126, and 444 mg/dL, respectively, whereas the respective minimum values are 57, 3, and 20 mg/dL. Blood samples were taken from all the subjects, and after clotting at room temperature these were then centrifuged at 3000 rpm for 10 minutes, and the supernatants were analyzed colorimetrically using a Hitachi 7600 analyzer. Ethical approval for this study was obtained from the Institutional Review Board of Cheng Ching General Hospital in Taiwan (IRB No: HP140014).

**Table 2:** **Baseline characteristics of lipid profile**

	TC	HDL-C	LDL-C	TG
	Whole set of the data (n = 3532)			
Mean	183.8	49.9	112.1	159.3
SD	40.1	15.3	34.3	110.3
Min.	57	3	20	22
Max.	569	126	444	1252
Q_1_	157	39	89	90
Median	181	48	110	130
Q_3_	208	58	132	192
	Cases with TG ≤ 400 (n = 3395)			
Mean	182	50.4	111.8	143.8
SD	38.4	15.3	33.6	73.6
Min.	57	3	20	22
Max.	395	126	312	399
Q_1_	156	40	89	89
Median	179	48	110	125
Q_3_	205	59	132	182
	Cases with TG > 400 (n = 137)			
Mean	227.4	38.6	119.2	544.8
SD	54.2	10.1	49.7	158.1
Min	120	8	36	401
Max	569	73	444	1252
Q_1_	191	33	88	438
Median	223	38	115	486
Q_3_	252	44	140	594

*TC* total cholesterol, *LDL-C* low density lipoprotein-cholesterol, *HDL-C* high density lipoprotein-cholesterol, *TG* triglyceride.

*SD* standard deviation, *Min.* minimum, *Max.* maximum, *Q1* 25% quartile, *Q3* 75% quartile.

All the units of lipid profile are mg/dL.

In summary, six groups were examined in this study based on age and gender: males 20–44 years old, 45–64, and 65 and above, and females in the same three age ranges. A total of 3,532 participants enrolled in the present study (2,152 men and 1,380 women).

### Measurement

Two approaches are typically employed to evaluate model adequacy. The first approach is to compare MSE, which measures the dispersion around the true value of the parameter. The lower the MSE value, the more accurate the formula. The second approach is to compare the underestimated and overestimated LDL-C values with the real values based on the existing formulae. An overestimate is defined as when the predicted value is greater than the true value whereas an underestimate is when the true value is greater than the predicted value.

## Results

This study carried out six experiments based on various combinations of age and gender. The results are presented in two parts, as follows:

### Study 1: Comparison of LDL-C MSE

**Figure 1:**
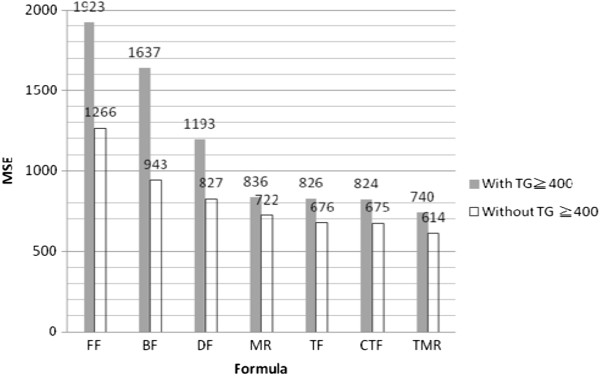
**MSE for all formulae with/without TG ≧400.** FF: Friedewald’s formula, BF: Balal’s formula, DF: DeLong’s formula, MR: Teerakanchana’s multiple regression formula, TF: Tsai’s formula, CTF: Calibrated from TF, TMR: Tsai’s multiple regression formula.

**Figure 2:**
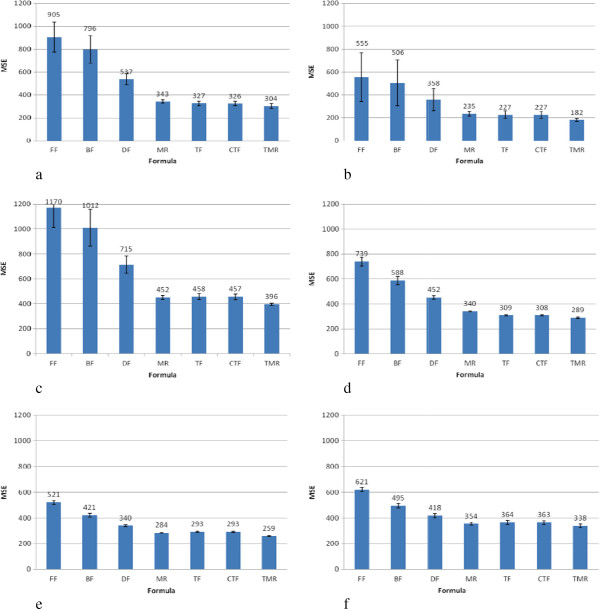
**MSE performance for genders in the different age groups. (a)** MSE for males in the younger group **(b)** MSE for females in the younger group **(c)** MSE for males in the middle-aged group **(d)** MSE for females in the middle-aged group **(e)** MSE for males in the elderly group **(f)** MSE for females in the elderly group. FF: Friedewald’s formula, BF: Balal’s formula, DF: DeLong’s formula, MR: Teerakanchana’s multiple regression formula, TF: Tsai’s formula, CTF: Calibrated from TF, TMR: Tsai’s multiple regression formula.

Figure 1 displays the MSE performance of all formulae with/without TG ≥ 400 mg/dL observations. These data show that the FF and BF that exclude TG ≥ 400 mg/dL have cutoffs of approximately 34% and 43%, respectively, while the other formulae are less affected by TG concentration. The FF is more accurate and precise when only observations with TG ≤ 400 mg/dL are considered [3]. In order to provide generalized benchmarking of the formulae, our experimental data include all levels of TG, i.e., all subjects were considered in the experimental analysis. The experimental results (Figure 2a- 2f) demonstrate that the FF has the largest MSE value, which indicates that it has the greatest differences between predictions and real observations. In fact, several studies have noted that the FF is known to underestimate the LDL-C concentration [4, 20]. In addition, the values predicted by MR, TF, CTF, and TMR, which have lower MSE values than the other formulae, are approximately half that of the vales predicted by the FF formula for both age and gender categories in our study. Furthermore, these four formulae also produce less variability in the error bars, and therefore less uncertainty in their predicted values (Figure 2a- 2f).

### Study 2: Comparison of LDL-C underestimation/overestimation

**Figure 3:**
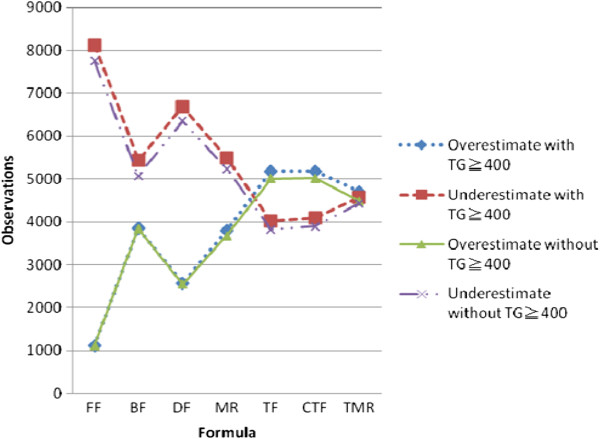
**Prediction performance for all formulae with/without TG ≧400.** FF: Friedewald’s formula, BF: Balal’s formula, DF: DeLong’s formula, MR: Teerakanchana’s multiple regression formula, TF: Tsai’s formula, CTF: Calibrated from TF, TMR: Tsai’s multiple regression formula.

**Figure 4:**
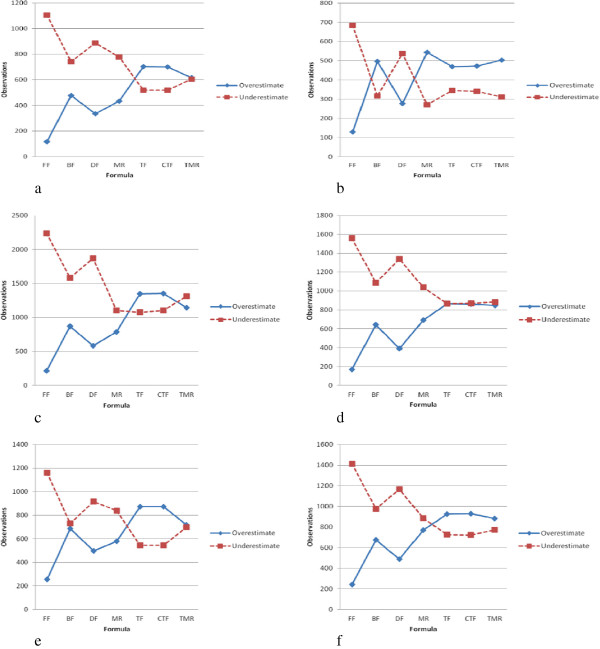
**Prediction performance for genders in the different age groups. (a)** Predictions for males in the younger group **(b)** Predictions for females in the younger group **(c)** Predictions for males in the middle-aged group **(d)** Predictions for females in the middle-aged group **(e)** Predictions for males in the elderly group **(f)** Predictions for females in the elderly group. FF: Friedewald’s formula, BF: Balal’s formula, DF: DeLong’s formula, MR: Teerakanchana’s multiple regression formula, TF: Tsai’s formula, CTF: Calibrated from TF, TMR: Tsai’s multiple regression formula.

Figure 3 shows the underestimated/overestimated performance of all formulae with/without TG ≥ 400 mg/dL. It is notable that the FF and BF have cutoffs of approximately 4% and 7% for the underestimated index without TG ≥ 400 mg/dL, respectively. As has been previously reported [3], FF is more accurate and precise when the observations only consider a TG ≤ 400 mg/dL. To provide generalized benchmarking for the formulae, our experimental data include all levels of TG. The dotted lines in Figure 4a- 4f represent an underestimated LDL-C prediction, i.e., when the predicted value is lower than the result of a medical test. The solid lines represent an overestimated LDL-C prediction, in which the predicted value is higher than the result from the test. These results show that the FF and DF tend to underestimate the LDL-C concentrations. These two formulae were the most consistent in terms of underestimating the LDL-C concentration in all six groups, and their predictions were affected by age and gender. BF and MR produced similar results to the FF and DF, in that they underestimated the LDL-C concentration in most cases. However, BF and MR provided fewer overall underestimated values compared to the FF and DF.

One finding of particular interest is that TF and CTF both produce not only similar numbers of observations but also produce more overestimates than underestimates (Figure 4a- 4f). Therefore, if there is a preference for patient safety by virtue of overestimated predictions of LDL-C, then TF and CTF can provide safer and more accurate results. That is, underestimates of LDL-C suggest that patients are in better health than they really are, while overestimates can provide an early warning for patients, so that they may choose to have more advanced medical tests performed.

Based on the MSE performance findings of this study, MR, TF, CTF, and TMR are preferable for estimating LDL-C concentration, as there is less variability in their results. We then assessed these formulae based on the degree to which they overestimate or underestimate the actual results. Tsai’s formulae, TF and CTF, performed the best in the LDL-C concentration estimations for all groups except females aged 20–44, with Teerakanchana’s multiple regression (MR) providing better results for this group. Moreover, TMR is a formula that can be easily applied to all groups, even though its performance among men and women aged 45–64 was slightly inaccurate than that of both TF and CTF, because these latter two approaches tend to underestimate the LDL-C concentration. Note that CTF is a revised version of TF, and if both formulae are recommended then TF should be used, because it is simpler than CTF. It is anticipated that clinical practitioners will be able to utilize the formulae benchmarking table produced in this work (Table 3), in order to choose the appropriate method for estimating LDL-C concentration when age and gender are taken into consideration.

**Table 3:** **Formulae benchmarking based on the cross-validation of age and gender**

	Gender					
	Male			Female		
**Age**	MSE	Under/Over	***Cross***	MSE	Under/Over	***Cross***
			Validation*			Validation*
20-44	MR	MR	***TF***	TF	BF	***MR***
	TF	TF	***CTF***	CTF	MR	***TF***
	CTF	CTF	***TMR***	TMR	TF	***CTF***
	TMR	TMR			CTF	***TMR***
					TMR	
45-64	MR	MR	***TF***	TF	TF	***TF***
	TF	TF	***CTF***	CTF	CTF	***CTF***
	CTF	CTF				
	TMR	TMR				
65+	MR	MR	***TF***	TF	TF	***TF***
	TF	TF	***CTF***	CTF	CTF	***CTF***
	CTF	CTF	***TMR***	TMR	TMR	***TMR***
	TMR	TMR				

Under/Over: Underestimate/Overestimate.

*MR* Teerakanchana’s multiple regression formula, *TF* Tsai’s formula, *CTF* Calibrated formula, *TMR* Tsai’s multiple regression formula.

*: The formulae benchmarking.

## Discussion

The results shown in Figures 1 and 2 indicate that the FF has relatively low accuracy. Although it exhibits relatively good performance among older women (aged 45 and above) with TG ≤ 400 mg/dL, its overall performance is worse than that of the other formulae. The formula with the best performance is TMR, followed by TF, CTF, and MR, with no significant differences among them, and the TF and CTF values in particular being virtually identical. Due to the properties of the multiple regression equation, the coefficients are more complex for MR and TMR. In terms of ease of use, TF is the preferred formula.

According to Tsai’s analyses, the FF tends to underestimate LDL-C concentration by 10.1 mg/dL on average [7], while Balal et al. [6] report that the FF underestimates it by 8 mg/dL, and other studies have shown similar results [14–18]. Tsai’s results also showed that the difference in the maximum and minimum for the FF is larger than that of the other formulae, and concluded that it is unsuitable for research on epidemiological or causal relationships [7].

For all cases examined with/without TG ≥ 400 mg/dL in this study, BF, the formula proposed by Balal et al. [6], provided better results than the FF, although it was still not as good as the other formulae. Tsai et al. [7] report that BF has exactly the same R^2^ as the FF, suggesting that BF only calibrated the underestimation of the FF. These results demonstrate that while the calibrated formula, acquired from the regression of the estimated value and the measured value, could produce an average estimated error that approaches zero and hence reduce the estimated bias, this still would not make the estimation more precise [7]. In addition, an LDL-C formula is primarily used to precisely estimate the LDL-C concentration for individuals, and while reducing the group estimated bias is important, this only reduces part of the individual estimated bias by expanding another part of it, and the standard deviation of estimated error is not improved. As shown in this study, BF is not able to replace the FF or improve its shortcomings.

As noted above, the best performance for the FF was in subjects with TG ≤ 400 mg/dL, although even among these it was outperformed by the other formulae, which provided stable results when age and gender were taken into account.

Based on a multiple linear regression analysis of 1,016 cases, Teerakanchana et al. [5] obtained the formula LDL-C = 0.910TC - 0.634(HDL-C) - 0.111TG - 6.755. Tsai et al. [8] also analyzed training data with multiple linear regression, and found that LDL-C = 0.9882TC - 0.8526(HDL-C) - 0.1065TG - 8.7029, with an R^2^ value similar to that of MR (R^2^ = 0.9649) and TF (R^2^ = 0.9608). In the present study, the R^2^ values for MR and TMR were determined to be 0.9648 and 0.9597, respectively; thus, there was no substantial difference between them in this respect. Since multiple linear regression analysis, TMR, is far more complex than TF, it is suggested that TF be used in most cases.

Because LDL-C tests tend to be time-consuming and inconvenient, the FF of LDL-C = TC - (HDL-C) - (VLDL-C) is often clinically applied to produce estimates of this value [3]. This formula assumes that the VLDL-C of healthy adults, except those with type III hyperlipidemia, is TG/5 [3, 23, 24] without chylomicrons. However, when using FF, VLDL-C would be overestimated, causing the underestimation of LDL-C, when TG chylomicrons and related remnants appear in plasma [25]. FF also assumes that TC only contains LDL-C, HDL-C, and VLDL-C, although it likely contains other constituents as well. For example, it has been shown that TC also contains intermediate-density lipoprotein cholesterol (IDL-C), chylomicrons, VLDL-C remnants, lipoprotein(a) [Lp(a)], Lp-X, and some fats that cannot be quantified with current methods [26]. In this case, when the contents other than HDL-C and LDL-C in TC are defined as RC, then RC = TC - (HDL-C) - (LDL-C) would be more accurate than using VLDL-C to estimate the RC. When TG has a specific relationship with RC, it would be more reasonable to estimate RC using TG [8]. When the regression analysis takes into account that TC contains LDL-C, HDL-C, VLDL-C, IDL-C, chylomicrons, Lp(a), Lp-X, and other non-quantifiable fats, Tsai et al. suggest revising the FF using TG = 1/8 instead of TG = 1/5 [8].

### Research limitations

In this study, participants with diabetes, secondary dyslipidemias (e.g., dyslipidemia due to renal, liver, or thyroid disease), and those who were taking statins or other lipid-modifying agents at the time of the enrollment were not excluded. In addition, the extrapolation of findings to other populations could introduce errors. The experimental benchmarking is therefore deemed specific for the Taiwanese cohort in this study.

Some subjects with heritable hyperlipidemia have extremely high TG. However, the current study had few cases with TG > 1500 mg/dL; these were not included in the analyses. In addition, some related studies were carried out after the subjects had fasted for 12 hours [27, 28], while in this study the subjects fasted for 8 hours, and this may have produced some discrepancies with previous results, which is an issue that requires further examination.

## Conclusions

Advances in current testing technology have resulted in efficient quantification of LDL-C concentration, although the costs of these technologies are relatively high. In contrast, estimating LDL-C concentration using formulae can produce reliable results at a relatively low cost, particularly when carrying out a large number of tests. We compared the results of direct homogeneous LDL-C assay with the FF, DF, MR, BF, TF, CTF, and TMR for determination of LDL-C based on underestimates/overestimates and MSE, using various combinations of age and gender. In terms of prediction errors and MSE, TF and CTF were the most accurate with regard to LDL-C concentration, except for women aged 20–44. Table 3 provides details for benchmarking the formulae when considering age and gender, and this could be a valuable reference for clinical practitioners deciding on the best estimation method for their particular situation.

## Abbreviations

FF: Friedewald formula
LDL-C: Low-density lipoprotein cholesterol
MSE: Mean squared error
TG: Triglyceride
CTF: Calibrated from TF
DF: DeLong’s formula
MR: Teerakanchana’s multiple regression
BF: Balal’s formula, which is calibrated from FF
TF: Tsai’s formula
TMR: Tsai’s multiple regression
TC: Total cholesterol
HDL-C: High-density lipoprotein cholesterol
VLDL-C: Very-low-density lipoprotein cholesterol.
